# Factors associated with poor sleep quality in women with cancer^1^

**DOI:** 10.1590/1518-8345.1478.2858

**Published:** 2017-03-09

**Authors:** Thalyta Cristina Mansano-Schlosser, Maria Filomena Ceolim

**Affiliations:** 2 Post-doctoral fellow, Faculdade de Enfermagem, Universidade Federal do Rio Grande do Norte, Natal, RN, Brazil. Scholarship holder from Coordenação de Aperfeiçoamento de Pessoal de Nível Superior (CAPES), Brazil.; 3 Associate Professor, Faculdade de Enfermagem, Universidade Estadual de Campinas, Campinas, SP, Brazil.

**Keywords:** Sleep, Breast Neoplasms, Depression, Nursing, Hope, Longitudinal Studies

## Abstract

**Objectives:**

to analyze the factors associated with poor sleep quality, its characteristics and components in women with breast cancer prior to surgery for removing the tumor and throughout the follow-up.

**Method:**

longitudinal study in a teaching hospital, with a sample of 102 women. The following were used: a questionnaire for sociodemographic and clinical characterization, the Pittsburgh Sleep Quality Index; the Beck Depression Inventory; and the Herth Hope Scale. Data collection covered from prior to the surgery for removal of the tumor (T0) to T1, on average 3.2 months; T2, on average 6.1 months; and T3, on average 12.4 months. Descriptive statistics and the Generalized Estimating Equations model were used.

**Results:**

depression and pain contributed to the increase in the score of the Pittsburgh Sleep Quality Index, and hope, to the reduction of the score - independently - throughout follow-up. Sleep disturbances were the component with the highest score throughout follow-up.

**Conclusion:**

the presence of depression and pain, prior to the surgery, contributed to the increase in the global score of the Pittsburgh Sleep Quality Index, which indicates worse quality of sleep throughout follow-up; greater hope, in its turn, influenced the reduction of the score of the Pittsburgh Sleep Quality Index.

## Introduction

Breast cancer is a disease which constitutes a serious public health problem, due to its high incidence and prevalence, as it is the type of cancer which most affects women worldwide. The estimate for Brazil for the 2016 - 2017 biennial indicates the occurrence of approximately 600,000 new cases of cancer, in which the epidemiological profile in women indicates breast cancer with 58,000 cases^1^.

Among the factors which negatively affect their quality of life, patients with breast cancer experience the presence of depression, anxiety, fatigue, pain and sleep disturbances - it being the case that these can also contribute to an increase in mortality^2^. The relevance of studies geared towards understanding these factors is undeniable, due to their complexity and to the impact they have on these women's health and daily life.

It is estimated that poor sleep quality is present in 85% of women with breast cancer, and that, in these women, it is shown to be associated with the presence of depression, low self-esteem, and pain^3^.

The high prevalence of poor quality sleep is concerning, as it is frequently found in association with worsening of health - affecting the regulation of the immunological and inflammatory functions, in the same way that it may cause changes in cognition and memory, emotional instability, and increase in appetite^4^. The management of poor quality sleep is important in these women, and should be preceded by the identification of the factors associated with it, at different points of the diagnosis and treatment.

A recent literature review, in patients who finished their treatment for breast cancer, demonstrates that they continue to experience some symptoms in the long-term: fatigue, depression, sleep disturbances and cognitive dysfunction. These symptoms often persist after the end of the treatment, resulting in a series of negative impacts on the patient's quality of life. This points to the relevance of follow-up surveys on these women for the better understanding of the interrelationship between these symptoms^5^.

Besides symptoms which negatively impact sleep quality, there are positive aspects such as hope, which can and must be encouraged in patients with cancer, constituting a strategy which can help the patient to cope with the disease and minimize the impact of adverse symptoms^6^ ^-^ ^7^.

This being the case, this study's objective was: to analyze the factors associated with poor sleep quality, its characteristics and components in women with breast cancer, prior to the surgery for removing the tumor, and throughout the follow-up.

## Methods

An analytical and longitudinal study, undertaken in a Women's Comprehensive Healthcare Center, with major coverage in the state of São Paulo, covering 42 municipalities and with nearly five million people treated each year.

The study had the following inclusion criteria: women aged 18 years old or over, with a diagnosis of breast cancer, TNM_0_ at any stage^8^, who were undertaking adjuvant chemotherapy and/or radiotherapy throughout the treatment, being treated in a hospital specialized in attendance to women, and receiving inpatient treatment due to mastectomy or quadrantectomy. The TNM system is the main system used in the staging of cancer, in accordance with Tumor (T), Node (N) and Metastasis (M); as an inclusion criteria, the researchers included those women in (0, 1, 2 or 3) and M0 as 'without metastasis'^8^. The exclusion criteria for the study were: Karnofsky Scale below 70 (the individual is able to care for herself for the majority of her needs, but these require a greater or lesser degree of dependence on the help of third parties); inadequate clinical conditions (such as mucositis, pain, nausea, dyspnea or vomiting) and inadequate emotional conditions (such as crying, apathy or aggression) for responding to an interview.

All the women receiving inpatient treatment due to surgery for removal of the tumor during the interval stipulated for data collection were included in the study, as long as they satisfied the selection criteria, totaling 156 participants at the beginning of the treatment (T0). None of the women approached declined to participate. These women were monitored over 12.4 months, on average, during the clinical treatment in the outpatient centers of the above-mentioned hospital. Due to the losses to follow-up (failure to appear for interview, deaths and incompleteness of data in the medical records), the study was undertaken with 102 women who completed all four stages of the study (T0, T1, T2 and T3).

**Figure 1:**
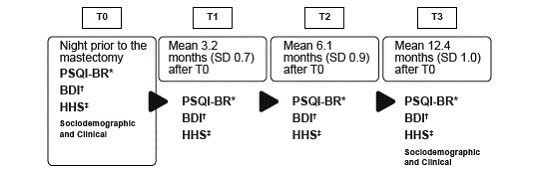
Description of the data collection times and instruments used in the women with breast cancer (n=102) Campinas, São Paulo, Brazil

The study was undertaken from March 2013 (the beginning of the baseline or T0) until December 2014 (end of data collection) of T3). The last participant was included in December 2013. The instruments used were the Sociodemographic and Clinical instruments (in T1 and T3); the Pittsburgh Sleep Quality Index (translated and validated for Brazil) (PSQI-BR), the Beck Depression Inventory (BDI) and the Herth Hope Scale (HHS), these at all points. The collection times are found in Figure 1.

The data collection instruments used were answered in the form of interview at the four points of the investigation, with the exception of the Sociodemographic and Clinical Characterization Questionnaire, which was used at the beginning and end of the study. These were, namely:

- Sociodemographic and Clinical Characterization Questionnaire: adopted based on a study undertaken in patients with cancer^9^ and subjected to content validation by specialists. This contains questions for sociodemographic and clinical characterization of the sample and was answered by the women and confirmed in the medical records by the researcher. In the medical records there were incomplete areas referent to clinical issues of the tumor such as the hormones estrogen and progesterone, or data on staging, which lead to loss to follow-up.

- The Pittsburgh Sleep Quality Index (PSQI-BR)^10^: validated in Brazil^11^. This allows the subjective assessment of sleep quality and problems throughout the month prior to the application of the questionnaire. It contains 19 questions, grouped in seven components: subjective sleep quality, latency, duration, efficiency, sleep disturbances, use of sleeping medication and daytime dysfunction. The global score varies from 0 - 21 points, and higher values correspond to worse assessment of sleep. When above five, it indicates poor sleep quality^11^.

- The Beck Depression Inventory (BDI)^12^: a self-assessment measurement of depression, broadly used in research and in clinical practice, validated in Brazil^13^. The original scale consists of 21 items, including symptoms and attitudes, whose intensity varies from zero to three. The items refer to sadness, pessimism, a feeling of failure, lack of satisfaction, feelings of guilt, feelings of punishment, self-deprecation, self accusation, suicidal ideation, bouts of crying, irritability, social withdrawal, indecisiveness, distortion of body image, work inhibition, sleep disturbance, fatigue, lack of appetite, weight loss, somatic preoccupation and reduction in libido. The following cut-off points were observed: below 10 - without depression, or with minimal depression; from 10 to 18 - mild to moderate depression; from 19 to 29 - moderate to serious depression; and 30 to 63 - severe depression^12^. Next, they were grouped into two categories: "without depression" and "with depression" (encompassing mild, moderate and severe depression).

- The Herth Hope Scale (HHS)^14^_,_ validated for use in Brazil^15^. This is made up of 12 statements with responses on a Likert-type scale (values of 1 to 4), with the following possibilities for response: disagree completely, disagree, agree, and agree completely. The total score varies from 12 to 48 points; the higher the score, the higher the level of hope^15^.

The treatment of the data was undertaken with the support of a statistician, and consisted of the descriptive analysis and construction of the Generalized Estimating Equations model (GEE)^16^, for the identification of factors present in T0 which influenced sleep quality throughout the follow-up period. A level of significance of 5% was considered. The analysis of reliability of the PSQI-BR was undertaken using the Cronbach alpha coefficient.

The ethical considerations were respected, in accordance with Resolution 466/2012, of the National Health Council, and the study was approved by the Research Ethics Committee of the institution to which this study's authors are affiliated, under Opinion N. 44169, CAAE 00762112.0.0000.5404 and its amendment was approved on 23^rd^ June 2015, under Opinion N. 1.106.951.

## Results

The 102 participants presented a mean age of 56.2 (SD 12.5) years old and stated an average of 5.3 (SD 4.0) years of study. Other sociodemographic and clinical data are provided in Table 1. The staging of the cancer was grouped in I/II as it is considered to be initial and constituted the majority of cases in this study.

**Table 1:** Sociodemographic and clinical characteristics of the women with breast cancer who participated in the study (n=102). Campinas, SP, Brazil, 2013-2014

Sociodemographic and clinical characteristics		N	%
Marital status			
	Has partner	56	54.9
Work situation			
	Retired	47	46.1
	Employed	23	22.6
	Unemployed	32	31.4
With whom patient lives			
	Family members	90	88.2
	Alone	7	06.9
	Others	5	04.9
Family income			
	Up to 5 minimum salaries*	93	91.2
	6 to 10 minimum salaries	9	08.8
Report of any other chronic illness			
	Yes	37	36.3
Symptoms related to the menopause			
	Yes	36	35.3
Report of pain			
	Yes	40	39.2
Staging (according to the TNM)**^†^**			
	I/II	83	81.4
	III	19	18.6
Neoadjuvant chemotherapy			
	Yes	26	25.2
Surgery undertaken			
	Mastectomy	57	55.9
	Quadrantectomy	44	43.1
	No information	01	01.0

*Minimum salaries in Brazilian reais R$ 724.00 Brazil, 2014; †TNM: Tumor (T), Node (N) and Metastasis (M).

Regarding the classification of depression in T0, 52.0% of the participants were in the category 'without depression or minimal depression', 18.6% had mild to moderate depression, and 29.4% had moderate or severe depression. The score for depression was identified as 11.2 (SD 9.2) on average. Hope, according to the HHS, obtained at T0 a mean score of 34.5 points (SD 6.3). The results of the descriptive statistics for the sleep characteristics, of the total score and all of the components of the PSQI-BR during follow-up are found in Table 2.

**Table 2:** Characteristics of sleep and components of the Pittsburgh Sleep Quality Index in women with breast cancer (n=102). Campinas, SP, Brazil, 2013/2014

	T0			T1			T2			T3		
	M*	SD**^†^**	Med**^‡^**	M*	SD**^†^**	Med**^‡^**	M*	SD**^†^**	Med**^‡^**	M*	SD**^†^**	Med**^‡^**
Sleep characteristics												
Duration (hours)	6.5	01.9	7.0	6.4	2.0	7.0	6.5	1.9	7.0	7.0	1.5	7.5
Efficiency (%)	95.8	27.6	94.0	86.3	23.5	86.0	87.5	24.7	89.0	88.4	19.9	89.0
Components of the PSQI§-BR												
Sleep quality	1.2	1.3	1.0	1.2	1.3	1.0	1.2	1.2	1.0	1.5	1.2	1.0
Latency	1.4	0.9	2.0	1.3	1.5	1.0	1.3	1.0	1.0	1.2	0.9	1.0
Duration	1.0	1.2	1.0	1.2	1.2	1.0	1.1	1.1	1.0	0.7	0.9	0.5
Efficiency	0.8	1.1	0.0	1.1	1.2	0.0	1.0	1.2	0.0	0.8	1.1	0.0
Disturbances	1.4	0.6	1.0	1.5	0.6	1.0	1.5	0.6	1.5	1.6	0.6	2.0
Use of sleeping medication	0.8	1.3	0.0	0.5	1.0	0.0	0.7	1.2	0.0	0.9	1.2	0.0
Daytime dysfunction	0.5	0.8	0.0	0.4	0.7	0.0	0.7	0.7	1.0	0.9	0.9	1.0
Total score of the PSQI-BR	7.1	4.4	7.0	7.3	4.7	6.5	7.4	4.8	6.5	7.3	4.3	7.0

*M: mean

†SD: standard deviation

MED: median

§PSQI- Pittsburgh Sleep Quality Index - Brazil (BR)

Poor quality sleep, estimated by the score of the PSQI-BR, was observed in 57.8% of the women in T0, 56.9% in T1, 55.9% in T2 and 61.8% in T3.

Table 3 shows the factors which influenced the final score of the PSQI-BR, identified with the Generalized Estimating Equations model.

**Table 3:** Factors which influenced sleep quality throughout the follow-up according to the Generalized Estimating Equations model. Campinas, SP, Brazil, 2013-2014.

Factors	Coefficient	Confidence Interval 95%		p-value
Age (years)	0.03	-0.04	-0.09	0.4108
Marital status (ref*: married)	0.54	-0.68	1.76	0.3892
Years of study (years)	0.09	-0.09	0.26	0.3235
Symptoms of the menopause (ref: no)	0.28	-0.99	1.55	0.6697
Staging of the tumor (ref: I or II)	1.25	-0.16	2.66	0.0822
Neoadjuvant chemotherapy (ref: no)	0.02	-1.47	1.51	0.9775
Dimension of the tumor (centimeters)	-0.13	-0.44	0.18	0.4009
Depression (ref: absent or minimal)	2.23	1.42	3.04	0.0001
Pain (ref: no)	1.31	0.01	2.62	0.0481
Score of the HHS**^†^** (score)	-0.08	-0.14	-0.02	0.0105

*Ref: indicates the reference category for the factor

†Herth Hope Scale

The presence of depression and complaints of pain presented a significant effect on quality of sleep throughout the follow-up, contributing to the increase in the score of the PSQI-BR. In the same way, lower scores of the HHS were related to the increase in the score of the PSQI-BR.

The analysis of the reliability of the PSQI-BR ascertained satisfactory results for the Cronbach alpha coefficient at the four points: T0 - 0.721, T1 - 0.782, T2 - 0.795 and T3 - 0.771.

## Discussion

Depression, pain and hope influenced sleep quality throughout the follow-up, with depression being the most significant factor in this study. In the literature, few investigations focus on the longitudinal monitoring^17^, considering that most researchers analyze cross sections in the different stages, and not the joint influence over the entire period^9^ ^,^ ^18^. Furthermore, data analyzed based on a longitudinal study after two years' treatment evidenced that the presence of some symptoms prior to the surgery had a predictive effect in the long-term on the quality of life of women with breast cancer, and the five symptoms present were: sleep disturbances, cognitive issues, physical tiredness, depression and anxiety. These authors concluded that it is necessary to assess symptoms in the pre-treatment period, in order to identify high-risk groups^19^.

In this study, the presence of depression and complaints of pain presented a significant effect on sleep quality over time, contributing to the increase in the score of the PSQI-BR. In one longitudinal study undertaken with 3343 women with breast cancer at an initial stage, evaluated 3 to 4 months after the surgery for resection of the tumor, the authors ascertained that depression was the strongest predictive factor for sleep alterations, a data which corroborates the present study^20^. Other researchers assessed 390 women with breast cancer prior to mastectomy and up to six months after, observing that more serious depressive symptoms were predictors of greater sleep alterations prior to the surgery, although this influence declined by the end of the follow-up^17^.

Coping with diseases which have poor prognoses, such as cancer, often entails the patient's psychological imbalance, and sometimes, in the daily routine of the services, there is no time for listening to the patient; also, the patient may feel discouraged from perceiving or talking about her feelings, distress or fear of death. There are specific instruments, as in the present study, which can be administered by health professionals for identifying such complaints and possible illnesses, such as depression^13^.

It should be highlighted that factors such as depression, for example, if not treated, may be present for years after the clinical treatment of the cancer^2^.

Compromising of sleep quality is considered to be a factor present in depression, so much so that one question regarding this forms part of the instrument for tracking depression used in this study. Authors have argued that the attempt to establish a unidirectional causal relationship might represent a simplification of an association which is in fact fairly complex, such that depressive symptoms can lead to poor sleep quality, and changes in sleep may contribute to the presence of depression in these women^20^.

Besides depression, pain was also a significant influence on poor quality sleep in this study. It is a frequent symptom in these patients, affecting 39.2% of the women in this study. High levels of depression, anxiety and sleep disturbances were present in women with breast cancer and who reported pain, in comparison with the group of women who did not have pain^21^.

Sociodemographic variables such as age and years of study were not significant in this study, in contrast with other authors, who showed that advanced age and fewer than seven years in full-time education were independent predictors of poor quality sleep^20^. In the present study, the majority of the women (75%) had been to school for fewer than eight years - and 50%, for fewer than four years, indicating greater homogeneity in this aspect, a fact which may explain the absence of results for this variable.

Poor quality of sleep was identified in 57.8% of the women at the beginning of the study, data which is similar to that of another longitudinal study with women with breast cancer, in which 57.9% of the women presented poor quality sleep^20^. A previous longitudinal study with 80 patients with breast cancer showed that poor quality sleep (PSQI ≥ 5) predominated at all points of the treatment (48.5-65.8%)^22^. In the present study, at the end of the follow-up, poor quality sleep persisted with an increase in the percentage of the women (61.8%) similar to that in a study with 166 women with breast cancer in which the results in the PSQI suggest that the women reported poor quality sleep prior to beginning treatment and mentioned even worse quality sleep after the end of the same^23^.

Regarding the components of the PSQI-BR, the component 'Sleep disturbances' obtained the highest score at all points, similar to the results found in another study monitoring women with breast cancer^24^. However, for these authors, the component 'Use of sleeping medication' obtained the lowest score, taking into account that in the present study it was 'Daytime dysfunction' that obtained the lowest score^24^. It should be emphasized that various aspects which participate in the component 'Sleep disturbances' are related to poor quality sleep in people with cancer, with emphasis placed on waking up early and the fragmentation of sleep, both for various reasons, such as the need to go to the toilet, pain, and worry^21^.

It stands out that high scores in the component of 'Sleep Disturbances' did not entail high scores in 'Daytime Dysfunction', suggesting that these women, although not sleeping well at night for the possible reasons mentioned above, did not complain significantly about the difficulty of remaining awake during their routine activities.

This study was guided by the need to identify factors which could be associated with poor sleep quality, as well as those which could contribute to its improvement. In a positive way - in this study - hope was shown to be effective for reducing the score of the PSQI-BR. It could, therefore, be used as a strategy by the health professionals for encouragement in coping better with the disease and the patients' day-to-day^7^.

Hope has been indicated as one of the resources for coping with breast cancer to be used in the practice of the health professionals, which could have positive consequences for sleep quality - although modest, as this study's findings suggest.

Based on this study's results, emphasis is placed on the need for longitudinal assessment of the quality and changes in sleep before, during and even after the treatment of the cancer, bearing in mind the persistence of poor quality sleep. In the same way, the relevance of planning and implementing interventions focusing on the modifiable factors which influence sleep quality, such as depression, pain and encouragement to hope, is surmised.

It is emphasized that the treatment of depression is known and that the identification of this threat to health is therefore necessary for it to be monitored and treated effectively. However, considering that hope constitutes a little-known factor, the need is evidenced for the health professionals to extend their knowledge in relation to it, for them to make use of the assessment instrument and seek a theoretical basis to make it possible to implant strategies in clinical practice. In the international literature, interventions encouraging hope are found in few studies - among which there is one investigation among carers of people with advanced cancer^(25)^, with results that the authors evaluated as satisfactory.

Further studies are necessary for assessing specific characteristics of the relationships between the factors identified in this study, and of the mechanisms for the management of the same which contribute to maintaining sleep quality or letting it worsen; and, furthermore, to ascertain whether there is a causal relationship rather than just of association between these variables, and the extent to which the treatment of depression and pain - and encouragement to hope - could contribute to improving assessment of sleep quality, in different stages of the treatment of the cancer, these necessarily being evaluated and treated by the health professionals.

As factors limiting the study, emphasis is placed on losses to follow up due to the women not attending, and to losses of data due to the lack of completeness of the medical records, reducing the sample size and the possibility of generalization of the results.

This study contributes to the advancement of the scientific knowledge of Brazilian and international Nursing, regarding the need for longitudinal assessment of sleep quality and the possibility of this in nurses' clinical practice or in the international journals which focus on modifiable influencing factors of sleep quality, such as depression, pain and encouragement to hope. Both the study and the knowledge of hope - as yet, studied but little in Brazil - might contribute as an innovation in Brazilian nursing science.

## Conclusion

The presence of depression and of pain prior to the surgery for the removal of the breast cancer contributed to the increase in the global score of the PSQI-BR, which indicates worse sleep quality, throughout the follow-up of the women in this study. The highest scores of the HHS, that is to say, the greatest hope, in their turn, influenced the reduction of the score of the PSQI-BR.

The persistence of poor quality sleep throughout the follow-up emphasizes the importance of assessing this parameter in patients with cancer, as well as of the relevance of the planning of interventions geared towards its improvement. This planning is only possible in conjunction with the identification of the factors which influence sleep quality.
